# Supplementary material for: Real-time, spatial decision support to optimize malaria vector control: The case of indoor residual spraying on Bioko Island, Equatorial Guinea
Source: PLOS Digit Health. 2022 May 12;1(5):e0000025. doi: 10.1371/journal.pdig.0000025 (PMC9931250; doi:10.1371/journal.pdig.0000025)
Supplement: S1 Text — The file provides more background on the logistics as well as some of the challenges of IRS operations on Bioko Island. (PDF) [file pdig.0000025.s001.pdf]

S1 Text: The need to establish a SDSS to implement IRS operations on Bioko Island

Here, we attempt to contextualize the implementation of the CIMS and the findings of the paper by describing historical challenges faced during IRS deployment on Bioko Island.

Urban spraying, household denominators and sprayer productivity

A high proportion of the implementation of IRS on Bioko takes place in high density and informal urban areas in the country capital, Malabo. Assessing true coverage over the first few years of IRS implementation was difficult due to changing household denominators from inconsistent counts, unknown deployment areas and the lack of a universal household identifier. The solution to this issue was brought about by the introduction of the grid-based coding system to enumerate houses and households [1] (S2 Fig). Additionally, sprayer productivity is influenced by the homeowner's availability and acceptance of IRS. In high population density areas, homeowners are often absent, thus reducing the time window of opportunity for spray teams to enter homes for IRS during working hours. Although there are many homes to spray in a targeted area, there also is a limited number of houses that can be sprayed per day and, thus, the perceived need to improve planning and deployment with the use of the CIMS.

Budgeting and planning

Planning IRS is based on available resources (*i.e.* budgets that take into account the cost of insecticide, labor and logistical operations) and need (*i.e.* malaria prevalence). The epidemiological situation of malaria determines the number of houses that *need to be sprayed*, but the budget determines the number of houses that *can be sprayed* in a given year. Importantly, the high cost of labor determines a time limit for ending a spray round, which means that any map-sectors undersprayed by that time will remain so. Hence, it becomes very important to utilize resources wisely and optimize IRS efficiency. This means that overspraying *here* may translate into underspraying *there*.

Selecting which houses to spray

The IRS deployment plan defines when and which map-sectors are sprayed. Within them, spraying is based on household acceptance and availability. The coverage target guides teams, but the coverage attained is highly determined by field conditions (*i.e.* how open are residents to accept spraying). Often, field teams struggle to obtain enough houses to reach the desired numerator. If and once the desired coverage is reached, teams are prompted by field managers to move on to the next map-sector in their deployment plan, and this is when

the SDSS plays a crucial role. If a household requests spraying, their house will always be sprayed, even if it happens to be outside the work plan. This, however, happens seldom.

Accessibility and community acceptance

Teams are also often forced to move on to the next map-sector in their deployment plan despite the target coverage not being attained. This usually happens due to refusals or houses being inaccessible. These factors are important drivers of underspraying in certain communities where residents recurrently refuse interventions. Data on availability and acceptability are captured through the CIMS and are used by field managers to make critical decisions during deployment (S4 Fig). The reasons why some residents recurrently reject IRS are the subject of active investigation by the BIMEP.

References

[1] García GA, Hergott DEB, Phiri WP, Perry M, Smith J, Osa Nfumu JO, et al. Mapping and enumerating houses and households to support malaria control interventions on Bioko Island. Malaria J. 2019;18(1):283.
