# Supplementary material for: Real-time, spatial decision support to optimize malaria vector control: The case of indoor residual spraying on Bioko Island, Equatorial Guinea
Source: PLOS Digit Health. 2022 May 12;1(5):e0000025. doi: 10.1371/journal.pdig.0000025 (PMC9931250; doi:10.1371/journal.pdig.0000025)
Supplement: S2 Text — The file contains technical details of the different components of the CIMS. (PDF) [file pdig.0000025.s002.pdf]

S2 Text: The CIMS infrastructure

The Campaign Information Management System (CIMS) was developed by Medical Care Development International (Silver Spring, MD, USA and Malabo, Equatorial Guinea) to support and manage the Bioko Island Malaria Elimination Project (BIMEP). Here, the CIMS components are explained in detail and further information is available at *https://github.com/cims-bioko*.

CIMS Server and CIMS Sidecar

Central to the CIMS is a robust database management infrastructure developed using open source PostgreSQL (The PostgreSQL Global Development Group) to configure and accommodate virtually all BIMEP field activities and campaigns. Every web-based, mobile, geospatial and analytic application hosted on the CIMS infrastructure employs PostgreSQL as their main data warehouse. The CIMS Server can be hosted on the Internet or on a local area network (LAN), and can be supported by the CIMS Sidecar, a zero-configuration service used to facilitate the synchronization of a lot of data from numerous Android tablets. Internet connectivity on Bioko is of limited bandwidth and the CIMS Sidecar bypasses this hurdle to favor data synchronization via LAN. A simplified workflow of the CIMS is shown in S1 Fig.

CIMS Forms and CIMS Mobile

Data entry forms are tailor-designed by field activity managers through CIMS Forms, a custom-built and open-source Android application forked from Open Data Kit (ODK) Collect (Get ODK Inc.). The forms, together with any associated ancillary data (*e.g.*, data on house locations and individuals, administrative level data, among others), are downloaded to mobile devices operating CIMS Mobile, an Android-based, open-source mobile application (Google Play, Google, LLC, CA, USA). Fieldworkers collect data offline onto CIMS Mobile using campaign tablets and upload the information back to the CIMS Server once they gain access to the Internet. Data on the CIMS Server are then processed and updated back to all connected mobile devices. The process is repeated within the campaign-specific parameters of a field activity. For example, forms cannot be uploaded after the end date of an activity. All data may be analyzed concurrently with data collection to provide feedback to fieldworkers, or they may be analyzed after the campaign has ended.

CIMS grid-based coding system

The coding system is based upon two overlapping grids of 1x1 kilometer *map-areas* and 100x100 meter *map-sectors* that are used to uniquely identify geographical features within and to guide fieldworkers without the necessity of geographical coordinates [1]. It was originally developed to map and enumerate all houses and households on Bioko Island to provide accurate denominators

and improve the management of malaria control activities (see more on denominator challenges, described below). A complete description of the system is available in [1] and is illustrated in S2 Fig. On Bioko, the CIMS relies on the coding system to provide the navigational hierarchy for fieldworkers to collect data using CIMS Mobile, but it is not a requirement in other settings. Furthermore, the coding system can be used to model and map any geographic feature beyond houses (*e.g.* mosquito breeding sites, health facilities, among others), and denominators for each geographic feature are updated during field campaigns, particularly catering for the fast-growing housing environment of Bioko Island [1]. All data collected during interventions are linked through this unique coding system that has grown to represent the basis for survey sampling and resource allocation.

The CIMS operational dashboards

The output from field activities collected via CIMS Mobile and processed through CIMS Sidecar and CIMS Server is represented through operational dashboards specifically designed for each activity and created using visualization software (Tableau Software, LLC, WA, USA). The ability to respond to real-time data is a requisite of any SDSS, and this is the final step within the CIMS infrastructure. The CIMS is considered a closed-loop system because data collection, processing and analyses are fed back to the field in real-time to support decision-making and prompt changes to the data coming back to the system. The dashboards are developed using data on metrics of productivity and coverage at various levels of geographical scale and granularity hosted in the CIMS Server. Productivity is tracked by showing the number of houses and rooms sprayed per day and the quantity of insecticide used per day. Coverage is displayed as houses and rooms sprayed against the recently updated denominator, population protected based on that day's household count and insecticide used as reported by the spray team. These data are ultimately visualized in maps at map-sector level (S3 Fig).

Capacity building for using the CIMS to support IRS operations and decision-making

Capacity building of staff in using data for decision-making has been a key activity of the BIMEP. All core field activities rely on teaching field workers to use the CIMS for planning, implementation and collection of data from field activities to interpreting the results for adaptively managing and optimizing the use of resources. Programme managers and data analysts oversee the data process and provide opportune feedback for decision-making. Real-time monitoring has proven instrumental for optimizing productivity and coverage. For example, during an ongoing campaign, whenever a map-sector reflects coverage equal to or higher than the programme target (80%), the teams are prompted to progress to the next map-sector in their respective deployment plan. Similarly, whenever productivity stalls, the dashboards aid the decision-making process for adjusting

deployment to map-sectors with available houses to spray in order to increase coverage. Moreover, reasons explaining drops in productivity are also investigated. For instance, map-sectors with low refusal rates are prioritized over those where sprayers find resistance in order to boost productivity (S4 Fig). The use of the CIMS has led to intensive training sessions and recurrent meetings with field staff to get them acquainted with the different needs and standards required for the proper operation of the SDSS. The process involves learning the technical aspects of using the system and, more importantly, understanding the basic concepts behind optimal coverage and operational efficiency.

References

[1] García GA, Hergott DEB, Phiri WP, Perry M, Smith J, Osa Nfumu JO, et al. Mapping and enumerating houses and households to support malaria control interventions on Bioko Island. Malaria J. 2019;18(1):283.
