# Supplementary material for: Real-time, spatial decision support to optimize malaria vector control: The case of indoor residual spraying on Bioko Island, Equatorial Guinea
Source: PLOS Digit Health. 2022 May 12;1(5):e0000025. doi: 10.1371/journal.pdig.0000025 (PMC9931250; doi:10.1371/journal.pdig.0000025)
Supplement: S1 Fig — (PDF) [file pdig.0000025.s003.pdf]

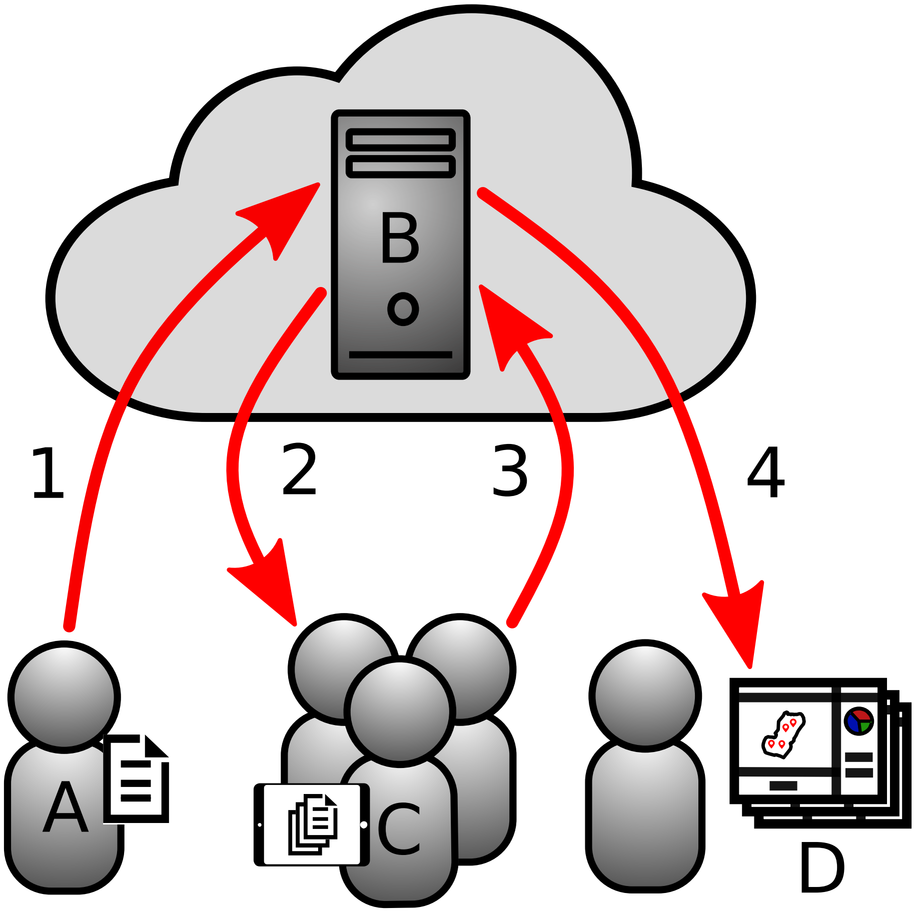

S1 Fig: A simplified schema of the workflow. A campaign manager (**A**) creates or updates a file that specifies how the system should implement the campaign. They upload this file (**1**) to the Server (**B**). Configuration and data are downloaded (**2**) to mobile devices by one or more fieldworkers (**C**). They use it to collect information in the field, where they may not have Internet access, using Mobile. Once they have Internet connectivity, fieldworkers (**C**) upload the collected data (**3**) back to the server (**B**), where they are processed. Collected data are downloaded (**4**) from the Server (**B**) to the dashboards (**D**), where they are used for decision support.
