# Supplementary material for: Real-time, spatial decision support to optimize malaria vector control: The case of indoor residual spraying on Bioko Island, Equatorial Guinea
Source: PLOS Digit Health. 2022 May 12;1(5):e0000025. doi: 10.1371/journal.pdig.0000025 (PMC9931250; doi:10.1371/journal.pdig.0000025)
Supplement: S2 Fig — (PDF) [file pdig.0000025.s004.pdf]

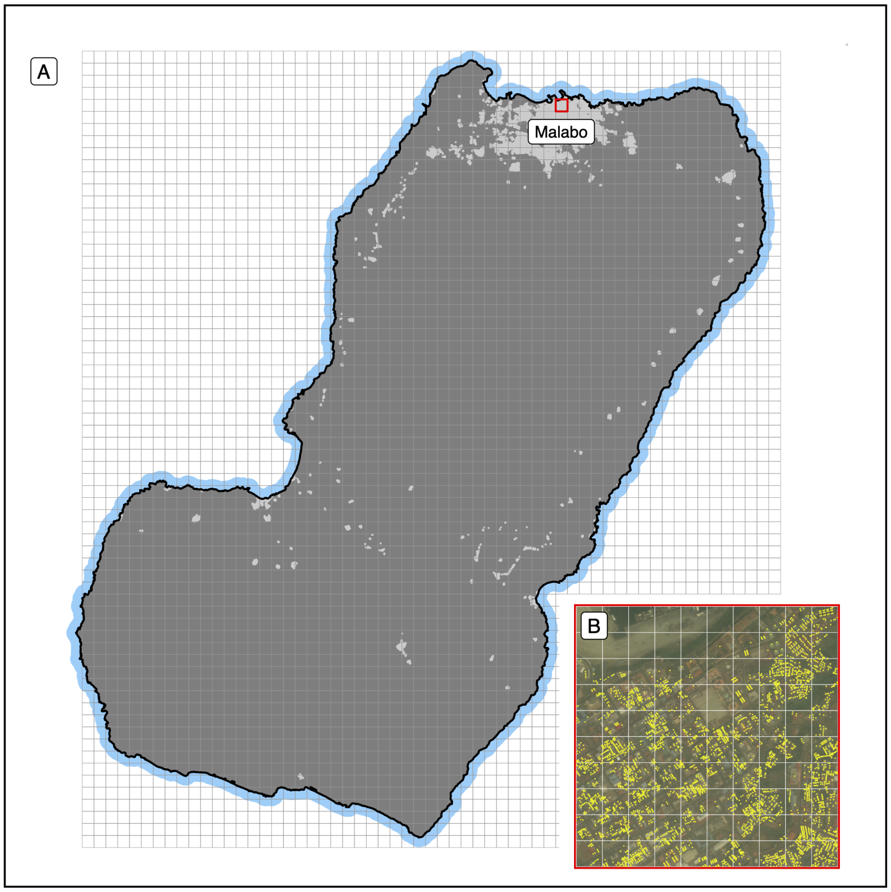

S2 Fig: The grid-based geographical coding system. **A.** The map-area grid overlaid on Bioko Island; each map-area is 1 by 1 km. The light grey dots represent houses, with the greatest concentration in the North, in and around Malabo, the country capital. **B.** Magnified view of the map-area marked in red in **A**. The map-sectors grid is shown overlaid on a satellite image and households indicated as yellow points to illustrate household density.
