# Supplementary material for: Real-time, spatial decision support to optimize malaria vector control: The case of indoor residual spraying on Bioko Island, Equatorial Guinea
Source: PLOS Digit Health. 2022 May 12;1(5):e0000025. doi: 10.1371/journal.pdig.0000025 (PMC9931250; doi:10.1371/journal.pdig.0000025)
Supplement: S3 Fig — (PDF) [file pdig.0000025.s005.pdf]

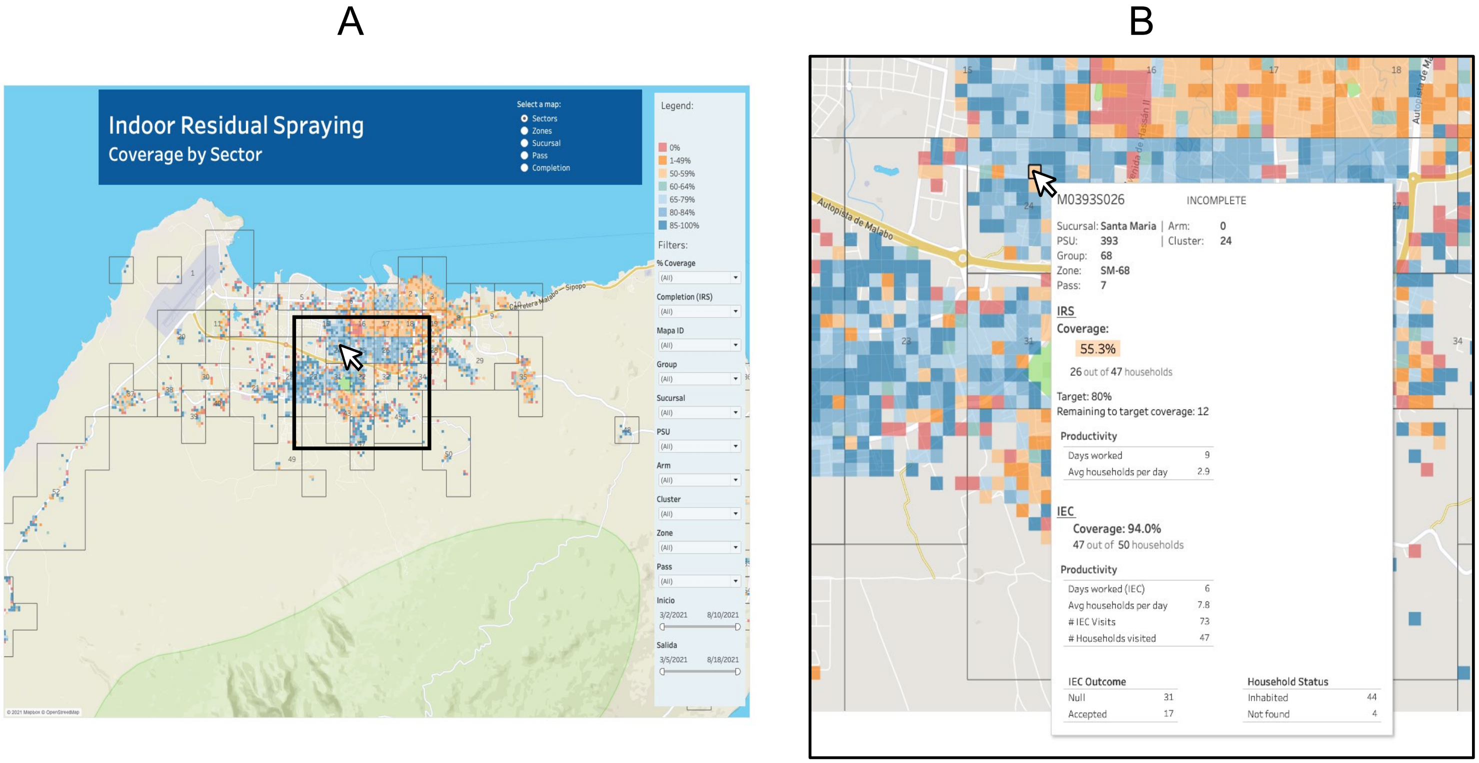

S3 Fig: Example of IRS coverage dashboard. Real-time maps are used to guide teams in the field. Map-sectors are color-coded according to coverage. **A.** Dashboard of coverage map including filters by administrative units and field team deployment plan. **B.** When the user clicks on any map-sector a pop-up box appears and displays relevant coverage and productivity indicators for that map-sector.
