# Supplementary material for: Real-time, spatial decision support to optimize malaria vector control: The case of indoor residual spraying on Bioko Island, Equatorial Guinea
Source: PLOS Digit Health. 2022 May 12;1(5):e0000025. doi: 10.1371/journal.pdig.0000025 (PMC9931250; doi:10.1371/journal.pdig.0000025)
Supplement: S4 Fig — (PDF) [file pdig.0000025.s006.pdf]

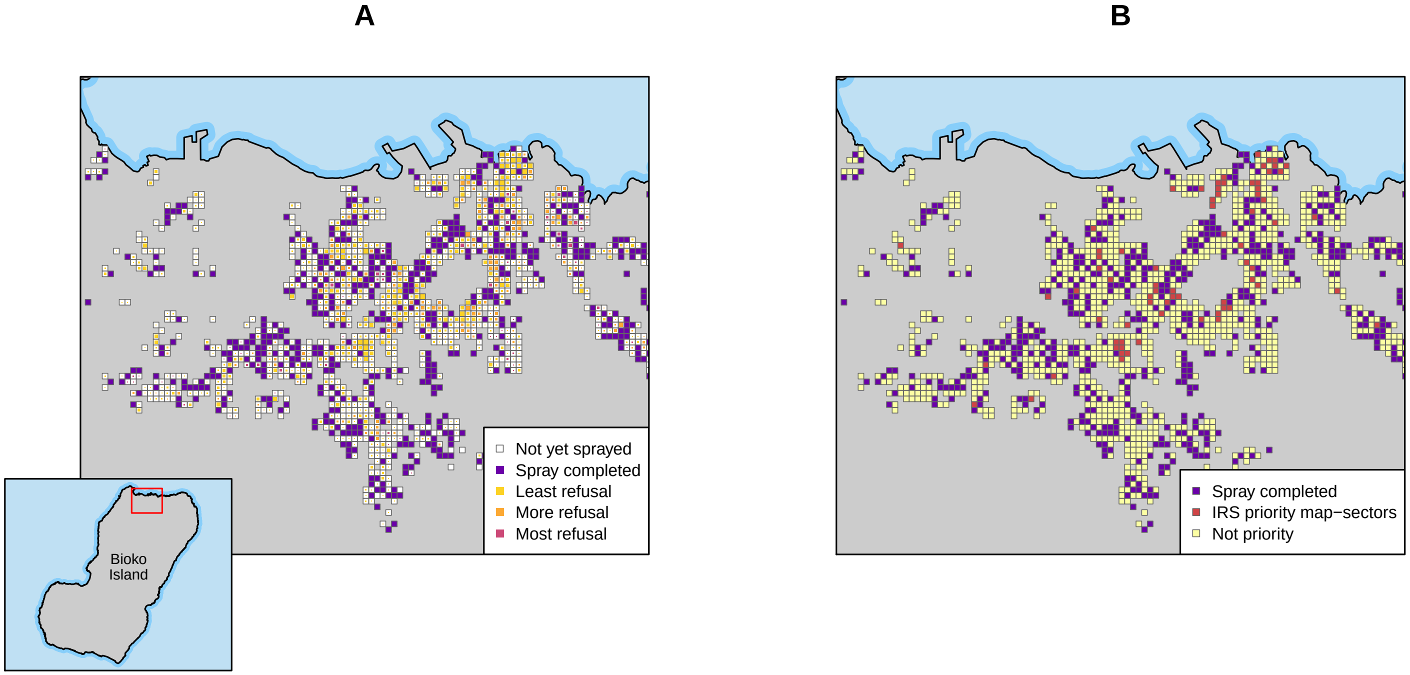

S4 Fig: Guiding based on refusals. Illustrative example of the 2020 round on Bioko Island, when field conditions were particularly challenging due to community refusal. Halfway through the campaign, map-sector-level data on coverage and refusal to the intervention were scrutinised in order to recommend priorities. **A.** The red square in the map inset, which corresponds to most of the capital city, Malabo, demarcates the area shown in the main panel. Map-sectors where coverage had been achieved were not considered further (purple pixels). The remaining map-sectors were classified according to the number of households left to spray, illustrated by the size of the pixel (the larger the pixel, the more houses left to spray), and the refusal rate, illustrated by the colour scale and expressed as the number of households refusing the intervention divided by the number of households left to spray in each map-sector (the redder the pixel, the higher the refusal rate). **B.** Priority map-sectors for were defined as those where there were more houses left to spray and where relatively few refusals were recorded. This approach helped guide the teams in a timely manner towards the areas where productivity and impact were expected to be highest.
