# Supplementary material for: Real-time, spatial decision support to optimize malaria vector control: The case of indoor residual spraying on Bioko Island, Equatorial Guinea
Source: PLOS Digit Health. 2022 May 12;1(5):e0000025. doi: 10.1371/journal.pdig.0000025 (PMC9931250; doi:10.1371/journal.pdig.0000025)
Supplement: Attachment — Submitted filename: PDIG-D-21-00081_reviewer.docx [file pdig.0000025.s007.docx]

Dear author:

I read your manuscript entitled: “**Real-time, spatial decision support to optimize malaria vector control: the case of indoor residual spraying on Bioko Island, Equatorial Guinea**”, and I understand that you using a spatial decision support system (SDSS) to handle the residual spraying on Bioko Island and to optimize malaria vector; and I will try to discuss the paper from a health geography point view, first of all, any geographic research is either:

- Thematic research focuses on how a natural, human or environmental phenomenon appears and spreads, and how is it linked to some geographical location and factors, as it depends mainly on the use of spatial, statistical, and geostatistical analysis tools.
- Territory management: for this approach the researcher used a specific technology to solve a territory problem (like malaria in your case), however, in this approach the problem is not important and researchers don’t focus on the situation and the problem (disease, flood, fires), but the aim is how to use the selected technique to solve this problem in a specific location.
- Methodological research is another level where the researchers try to produce new methods, tools, approaches or optimize the existing ones.

For your paper I suppose it’s A territory management paper, because the paper doesn’t discuss malaria or the causes of contamination and the spatial analysis of malaria, but it discusses “How to handle the malaria situation” so I will go with this approach and here are the suggested notes to make your article better (by the way, it’s a good idea and you make a great academic contribution), most of the notes are for formatting.

- Make your abstract more in-depth and more technical.
- You can insert a literature review about using SDSS in health, and some background about malaria disease and the spray operation.
- You have to make the problem more explicit.
- Explain technics and methods used in the paper in depth especially the architecture of the spray operation, software, tools, server, phone and user side…etc.
- Your result is more like a simple text and just describes and rewrites the results and table without any further technical and statistical details.
- Please rewrite your article using IMRAD structure (introduction, method, result, and discussions), I see that you put the result just after the introduction and you push the data and methods section to the end of the article, I think this is not suitable for an academic article.

Finally, please check the attached PDF file, I include some notes and comments. Good luck!
