# Supplementary material for: Real-time, spatial decision support to optimize malaria vector control: The case of indoor residual spraying on Bioko Island, Equatorial Guinea
Source: PLOS Digit Health. 2022 May 12;1(5):e0000025. doi: 10.1371/journal.pdig.0000025 (PMC9931250; doi:10.1371/journal.pdig.0000025)
Supplement: Attachment — Submitted filename: Response_reviewers.docx [file pdig.0000025.s009.docx]

**Reviewer #1:** This paper is technically satisfactory as far as it goes but in my opinion falls far short of what might be expected for a paper dealing with the development of a spatial decision support system (SDSS).  It might be fairer to say that this paper focuses simply on the development of a GIS to manage a spraying programme.

We are concerned by this confusion, as the CIMS definitely is not only a GIS to manage spraying but a SDSS. This perhaps derives from lack of clarity of our paper in conveying the message. We hope our answers below and the editorial changes to the paper clarify the reviewer’s confusion.

1.  I would like to see more discussion of the benefits derived from different spraying scenarios at the map sector level given the apparent spatial spillover effects associated with spraying (as remarked by the authors).  The system might be used to test these spatial spillover benefits and how different geographical patterns of spraying might be used to provide the most cost effective treatment.  This leads to my second and related point:

Our group is currently addressing questions precisely related to this topic, and this is the reason why this was not central to the current paper but rather ongoing work that we hope is submitted soon to peer-review. The questions include: What is the optimal coverage to provide community protection (in the paper, we assume that this lies within the 80 to 85% band, but acknowledge that this coverage could well be below - or above - this band)? What are the likely effect sizes of spill over derived from IRS? Our SDSS allows us to test scenarios like the ones presented in Fig 1 with real world data. This work is using a comprehensive modeling framework that is currently under development in collaboration with expert groups. We have added a mention of this ongoing work in the Discussion.

2. This paper confuses outputs and outcomes.  The purpose of a spraying programme is to reduce malaria (outcome) not simply to manage a spraying programme (output).  For this reason the system needs to include malaria data at the finest available space-time scale so that some attempt can be made, within the SDSS, to evaluate the effectiveness of any programme in relation to the outcome which is the aspect of this that matters.  This of course presents a significant evaluative challenge but that is what an SDSS is meant to address - otherwise it might just as well be called a GIS which is constructed for the sole purpose of managing and displaying spatial and spatial-temporal data about where and when spraying was carried out.

We agree with the reviewer that there is a difference between outcomes and outputs in the context of SDSS. Our paper strictly focuses on outputs on the operational side because we were concerned with process indicators rather than impact indicators. It is obvious that impact is the ultimate goal of any malaria control operation, but, often, the performance of processes (actual doings in the field) is ignored, introducing noise to indicators of impact.

As part of the BIMEP, we are striving to comprehensively monitor and evaluate malaria control on Bioko through the SDSS (CIMS), and this includes both the ends and the means. Our paper is telling one part of this story. The other part is currently the subject of analyses that aim to describe the hugely complex interactions of multiple factors that, despite control measures in place, are still driving malaria transmission on the island. We see the current paper as a stepping stone towards the grander goal of bettering our understanding of malaria epidemiology and control on Bioko Island and beyond.

We apologize for not conveying the aims of our paper clearly in the first version. We have re-written the aims in the last paragraph of the Introduction hoping this brings clarity.

As a final point more attention needs to be paid to the maps in Figure 1.  The choice of colour palette makes it very difficult for the reader to see where the different types of households are located.

We appreciate the recommendation. We have redone Figure 1 giving more contrast between sprayed and not sprayed houses.

**Reviewer #2**: Dear author:

I read your manuscript entitled: “Real-time, spatial decision support to optimize malaria vector control: the case of indoor residual spraying on Bioko Island, Equatorial Guinea”, and I understand that you using a spatial decision support system (SDSS) to handle the residual spraying on Bioko Island and to optimize malaria vector; and I will try to discuss the paper from a health geography point view, first of all, any geographic research is either:

•       Thematic research focuses on how a natural, human or environmental phenomenon appears and spreads, and how is it linked to some geographical location and factors, as it depends mainly on the use of spatial, statistical, and geostatistical analysis tools.

•       Territory management: for this approach the researcher used a specific technology to solve a territory problem (like malaria in your case), however, in this approach the problem is not important and researchers don’t focus on the situation and the problem (disease, flood, fires), but the aim is how to use the selected technique to solve this problem in a specific location.

•       Methodological research is another level where the researchers try to produce new methods, tools, approaches or optimize the existing ones.

For your paper I suppose it’s A territory management paper, because the paper doesn’t discuss malaria or the causes of contamination and the spatial analysis of malaria, but it discusses “How to handle the malaria situation” so I will go with this approach and here are the suggested notes to make your article better (by the way, it’s a good idea and you make a great academic contribution), most of the notes are for formatting.

We are grateful for the positive feedback.

•       Make your abstract more in-depth and more technical.

We have reconsidered the Abstract but feel that it provides a good summary of the methods and the findings. We have made minor amendments to improve the clarity of the aims of the paper.

•       You can insert a literature review about using SDSS in health, and some background about malaria disease and the spray operation.???

The first paragraph of the Introduction defines SDSS and the sixth paragraph of the Introduction presents a brief historical account of the evolution of SDSS to support malaria epidemiology, referencing the work of others. We felt that these accounts provide a concise background and guide the reader to what others have done. With regards to the spray operation and the CIMS itself, we have expanded the text in the Supplementary Information to provide additional information on both.

•       You have to make the problem more explicit.

The introduction and supplementary sections have been revised to make the problem of denominators and productivity more explicit, particularly as it relates to Bioko.

•       Explain technics and methods used in the paper in depth especially the architecture of the spray operation, software, tools, server, phone and user side…etc.

The Methods section now contains a description of the SDSS and the technical components of it. It is also explained at length in the Supplementary Information. As indicated above, we have expanded on the information contained in this document and we hope that the reviewer finds answers to his questions within. We feel that having this information in the main text would divert the reader from the main focus.

•       Your result is more like a simple text and just describes and rewrites the results and table without any further technical and statistical details.

We feel that the Results section does include the statistical significance of the primary indicators we established to compare across the 5 years of the data: coverage, operational efficiency, and productivity. The statistical analysis is also already described in the Methods.

•       Please rewrite your article using IMRAD structure (introduction, method, result, and discussions), I see that you put the result just after the introduction and you push the data and methods section to the end of the article, I think this is not suitable for an academic article.

We followed PLOS guidelines for a research article. We request editorial advice regarding which format is expected and are happy to go either way (i.e. Methods before the Results or after the Discussion).

Finally, please check the attached PDF file, I include some notes and comments. Good luck!

**Reviewer #3**: The authors present an interesting application of a SDSS to support an IRS implementation.

I was left a little confused by the aims of the paper: is it to “Describe the use of a novel SDSS to support malaria vector control?” (lines 49 to 50)?  Or does the paper present changes to IRS overtime as reported within a bespoke SDSS?

We apologize for the confusion. The aim of the paper was not to simply describe the use of the SDSS but rather how the use of the SDSS has impacted operational efficiency during IRS operations. We have re-written the aim in the first sentence of the last paragraph of the Introduction. We hope that this addresses the Reviewer’s confusion.

If it is the former, I was underwhelmed as to how the SDSS was actually used to support the IRS operations. I think more detail and a clearer picture on how the system is actually used would be of interest to the reader, rather than simply a presentation of the changes in “over-sprayed” and “under-sprayed” map-sectors.

Again, we apologize for the lack of clarity in the original version. We used the Supplementary Information to describe the technical details of the SDSS to keep the paper concise. We have made this more explicit in the Methods by directing the reader to the SI for more detailed information. We have also added information to the SI.

Some specific areas where more detail on how the SDSS is actually used include:

The abstract reads “High spatial granularity during planning and deployment together with closer follow-up of field teams using real-time data supported more homogeneous delivery of optimal coverage while sustaining high productivity.”, however in the main text of the manuscript I have failed to read or understand how the SDSS was practically used to support an IRS operation. Further clarification (in perhaps the Materials and Methods and the Discussion) would be of interest.

The Discussion refers to real-time monitoring through online dashboards (line 158). How were these dashboards used in an SDSS to support the IRS operation? Was this a standardized process incorporated into operation based on the SDSS? Can the reader see a snapshot of what the real-time dashboard monitoring interface looks like? Some detail in the Materials and Methods would be informative in regards to monitoring IRS operations using the SDSS.

I was left confused about a number of aspects of the SDSS and found the materials and methods section lacking sufficient detail to understand the operational processes involved in using the SDSS to support IRS:

•       How are baseline household data recorded and updated into the SDSS?

The island household denominator is constantly updated to adapt to the actively changing housing environment on the island. Every time a house is visited during any household intervention (i.e. MIS, IRS, bed net distribution) its status is updated. This is mentioned in the SI.

•       How are IRS data updated into the SDSS? The paper states it is real-time monitoring? How are these data reported to the SDSS (via mobile device? By the Spray Teams?)

This has been clarified further both in the Methods and in the SI.

•       What additional attribute data are recorded into the SDSS?

Data recorded in the CIMS include household status, household and housing characteristics, acceptance or refusal to interventions, reasons for refusals, etc.

     o  Is there any information collected or reported as to why households are not sprayed?

As per above, yes, this is part of the data recorded during IRS visits.

•       Can the grid-based coding system be described in a little more detail?

The grid based system is described in detail in the SI and full details were published in “García, G. A. et al. Mapping and enumerating houses and households to support malaria control interventions on Bioko Island. Malar. J. 18, 283 (2019)”. This paper is cited both in the main text and the SI as a reference for the grid system.

•       I believe some of this is described in the supplementary material and may be referenced, however for context I think some of this information should be included in the main text also.

Does the SDSS have capacity to facilitate follow-up spraying operations in under-sprayed areas as a means to improve coverage and achieve optimal outcomes?

As is mentioned above, we expanded on these details within the SI to keep the paper more concise. We have made a clearer reference to the document in the Introduction and Methods.

Also – who is the user of the SDSS? Field Managers? Overall Supervisors? Other people? Is there a high level of acceptability of the SDSS among the intended users?

This is addressed now in the final section of the SI, where we discuss about users of the SDSS and the learning process required to use the system.

It is an interesting argument in the Discussion that “operational efficiency increased significantly between 2017 to 2021 and that this improvement was attained by “significantly reducing overspraying” (Lines 127 to 129).  This seems like somewhat of a controversial and perhaps questionable operational decision – particularly when discussing “equitable” delivery of interventions??

•       How does a spray-person decide whose house not to spray within a map-sector? Surely this could create operational issues within a community? If a household requests spraying – are they given this intervention?

•       Is there evidence to suggest that over-spraying in certain “map-sectors” contributes to under-spraying elsewhere? And why so? Is it just simply a limited lack of resources and they run out of “insecticide”, or could there be other underlying factors inhibiting spray coverage in certain areas?

We appreciate these concerns. IRS is given based on a preset coverage target that defines the number of houses to be sprayed per sector, but this does not mean that if a resident requests spraying they do not receive the intervention. We strive to balance willingness to accept spraying, coverage targets and resources. Resources are limited in time, human power and commodities, hence overspraying here sometimes means under spraying there.

IRS on Bioko Island is bound to some particular challenges that we have now mentioned in the main text and Supplementary Information to provide context and address the Reviewer’s questions. We rewrote the Supplementary Information and added an account of IRS operations on Bioko and the main challenges faced. The SI is referenced repeatedly within the main text when such questions may arise.

•       Are there spatial patterns to underspraying? i.e. areas consistently undersprayed? Were data collected in regards to why certain areas were undersprayed? Can this information be accessed via the SDSS?

Indeed. Some residents recurrently refuse interventions and this happens more often in certain communities. This information is captured by the SDSS and patterns are actively sought to adapt interventions accordingly. This is one of the reasons why attaining optimal coverage to provide community protection becomes a very important goal. We have expanded on this topic in the new section of the Supplementary Information and have added Figure S4, which illustrates a specific example on the use of the SDSS in response to community refusals.
